# Draft genomes of 12 *Bifidobacterium* isolates from human IBD fecal samples

**DOI:** 10.1128/mra.00130-23

**Published:** 2023-12-15

**Authors:** Cole E. Souza, Nicole E. Jacobson, Michelle A. An, Lindsay Droit, Alejandro A. Vega, Mariel Rosales, Kathie A. Mihindukulasuriya, Karina Pastrana, Scott A. Handley, Miles Parkes, Joanna Rimmer, David Wang, Elizabeth A. Dinsdale, Robert A. Edwards, Anca M. Segall

**Affiliations:** 1 Department of Biology, Viral Information Institute, San Diego State University, San Diego, California, USA; 2 Department of Pathology and Immunology, Washington University School of Medicine in St. Louis, St. Louis, Missouri, USA; 3 Department of Medicine, University of Cambridge, Cambridge, United Kingdom; 4 Department of Medicine, Division of Gastroenterology, Addenbrooke's NHS Trust Hospital, Cambridge, United Kingdom; 5 Academic Department of Military Medicine, Royal Centre for Defence Medicine, Birmingham, United Kingdom; 6 Department of Microbiology, Washington University School of Medicine in St. Louis, St. Louis, Missouri, USA; 7 Flinders Accelerator for Microbiome Exploration (FAME), College of Science and Engineering, Flinders University, Bedford Park, South Australia, Australia; Loyola University Chicago, Chicago, Illinois, USA

**Keywords:** bifidobacteria, inflammatory bowel disease, genome sequences, fecal samples, human microbiome, gut microbiome

## Abstract

Twelve *Bifidobacterium* strains were isolated from fecal samples of inflammatory bowel disease patients and matched “household control” individuals. These include the species *Bifidobacterium adolescentis*, *Bifidobacterium animalis*, *Bifidobacterium breve*, *Bifidobacterium catenulatum*, *Bifidobacterium longum*, and *Bifidobacterium pseudocatenulatum*.

## ANNOUNCEMENT

Bacteria and bacteriophages play an integral role in our gut health (1, 2). Of specific interest is the *Bifidobacterium* genus of protective microbes, Gram-positive obligate anaerobes found ubiquitously in the animal and human gastrointestinal tracts, insect intestines, and the oral cavity (3). Patients with allergies, diabetes, and obesity have reduced *Bifidobacterium* numbers (4). In the context of inflammatory bowel disease (IBD), *Bifidobacterium* appears to protect the host against pathogens and inflammation by modulating the intestinal mucosal barrier and maintaining colonization resistance (5). The abundance of *Bifidobacterium* strains is reduced in patients with IBD compared to healthy individuals (6). Studies in animals and humans showed that *Bifidobacterium longum* can reduce colitis and chronic inflammation symptoms (7, 8).

Human fecal samples from patients with Crohn’s disease (CD) or ulcerative colitis (UC) and from healthy “household control” (HHC) individuals who live in the same household were collected at Addenbrookes NHS Trust Hospital in Cambridge, UK. These strains were isolated from stool samples collected under IRB# UK 05/Q0108/355 (University of Cambridge) and transferred to the US under IRB# 201910072 (Washington University in St. Louis).

From these samples, 12 of the sequenced bacterial isolates were *Bifidobacterium* spp. (Table 1). Human fecal samples (0.7–1 g each) were homogenized in 10 mL of SM buffer [100 mM NaCl, 8 mM MgSO_4_·7H_2_O, and 50 mM Tris-Cl (pH 7.5)]. From each sample, we isolated six to nine strains at 37°C in an anaerobic chamber using LB or BHIS [per liter: 38 g BHI, 5 g yeast extract, 1.62 mL of 300 mM MgSO_4_·7H_2_O, and 22.52 mL of 20 mM CaCl_2_ supplemented with menadione (K1) and hemin at a final concentration of 0.05%]. After three sequential rounds of streak isolation in the anaerobic chamber at 37°C, individual colonies were saved as 12.5% glycerol stocks at −80°C. Genomic DNA was prepared using the hexadecyltrimethylammonium bromide (CTAB) protocol (9).

**TABLE 1:** Assembly statistics for *Bifidobacterium* strains isolated from CERVAID IBD fecal samples

Strain ID	Species	Fecal sample	Total no. of reads	No. of contigs	N50	Contig length (nt)	% GC content	Annotated genes	NCBI accession
RC1_18990	*pseudocatenulatum*	UC	2,679,075	27	396,565	2,290,342	59.2	2,048	JARJNE000000000
RC3_18990	*longum*	UC	2,422,937	75	71,540	2,216,139	59.3	1,941	JARJNF000000000
RC7_19002	*adolescentis*	UC	2,024,287	29	122,711	2,209,182	59.4	2,178	JARJNG000000000
RC9_19002	*longum*	UC	2,708,185	50	104,221	2,249,948	60.2	1,996	JARJNH000000000
RC10_19003	*adolescentis*	HHC	2,619,984	31	178,914	2,237,269	59.8	1,972	JARJNI000000000
RC12_19003	*adolescentis*	HHC	2,407,369	30	153,912	2,272,964	59.7	2,041	JARJNJ000000000
RC28_18993	*longum*	HHC	1,476,172	41	151,627	2,390,673	60.2	2,155	JARJNK000000000
RC30_18993	*longum*	HHC	1,685,895	41	132,966	2,392,031	60.2	2,161	JARJNL000000000
RC92_19017	*catenulatum*	UC	1,765,824	44	141,994	2,069,620	58.7	1,844	JARJNM000000000
RC93_19017	*catenulatum*	UC	2,056,238	42	141,912	1,989,289	59.2	1,778	JARJNN000000000
RC150_19034	*animalis*	HHC	1,429,131	77	39,860	1,799,235	59.9	1,640	JARJNO000000000
RC158_19046	*breve*	CD	7,175,573	20	324,194	2,336,174	58.6	2,127	JARJNP000000000

**Fig 1:**
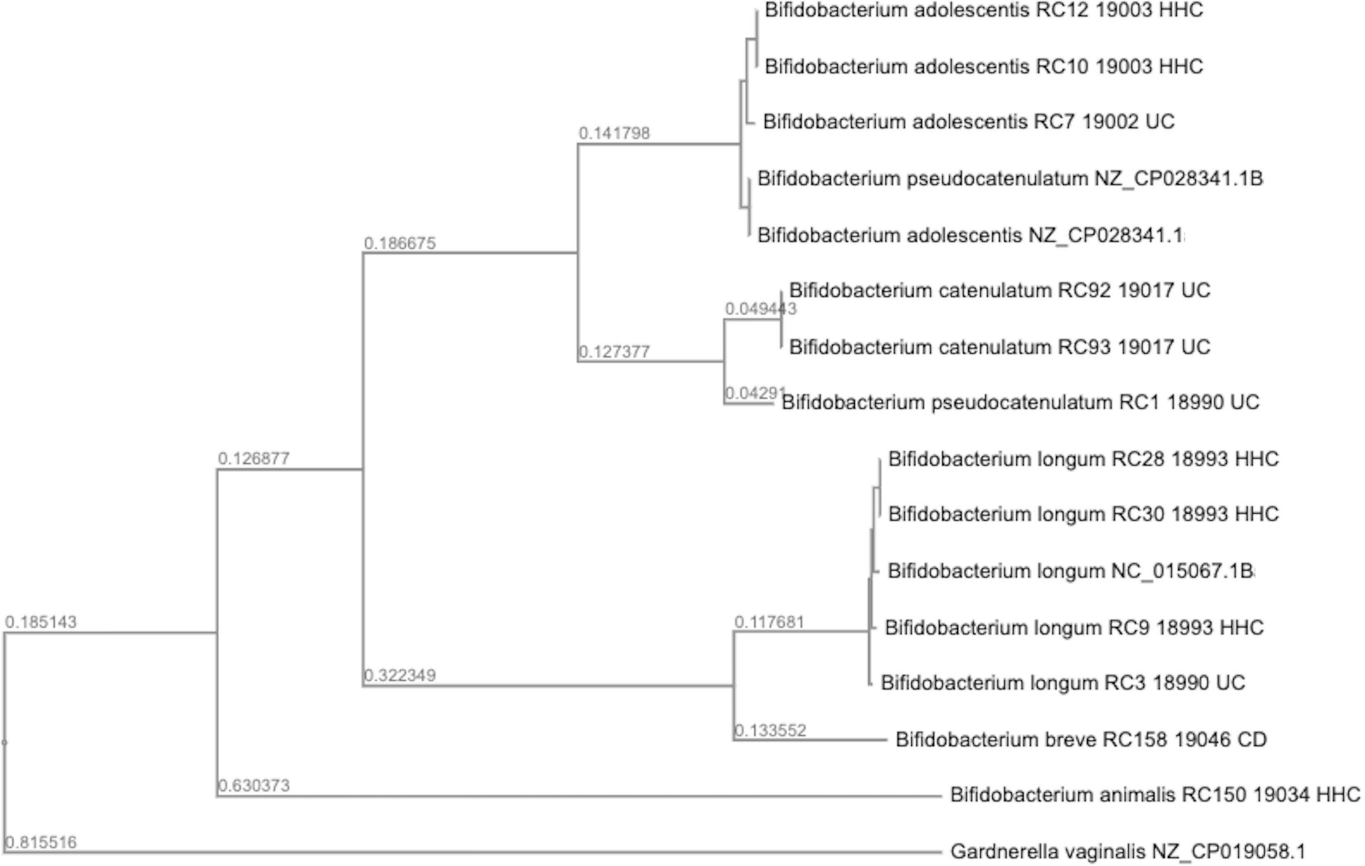
The tree was generated using the BV-BRC codon tree, which aligns 1,000 genes from different genomes to one another using the program RAxML (13). Here, we show *Bifidobacterium* strains isolated from IBD fecal samples, publicly available strains of *Bifidobacterium* as references, and the closest related Bifidobacteriaceae species. The Segall Lab strain designation, patient sample designation, and sample source (CD, UC, or HHC individual) are listed.

Genomic libraries were prepared using Nextera DNA Flex library kits and sequenced with NextSeq2000 by SeqCenter (https://www.seqcenter.com/; Table 1). Genomes were assembled using Unicycler version 2022 with default parameters. Taxonomic classification was determined using BLASTN of a segment of ~100,000 nucleotides from each assembled genome (10). A maximum likelihood-based phylogeny can be found in Fig. 1 (11). Genome annotation by NCBI used the NCBI Prokaryotic Genome Annotation Pipeline version 6.4. Given the average *Bifidobacterium* genome size of ~2.2 Mb, some genomes may be incomplete. All the genomes presented contain plasmid-like contigs with annotated ParA and ParB partitioning proteins and chromosome segregation ATPases; some include open reading frames (ORFs) annotated as integrase, transposase, and other proteins associated with mobile elements. Analysis of these *parA*- and *parB*-coding contigs with the machine learning tool PhANNs, which sensitively detects phage-associated structural proteins [(12); https://phanns.com/], indicated tail fiber, portal, and major capsid proteins. Hence, the plasmids present in these strains may be plasmid phages similar to *Escherichia coli* phage P1.

## Data Availability

Genomes are available at NCBI GenBank and sequences are present in the Sequence Read Archive (SRA) using the accession numbers shown (Table 1) as well as under the BioProject PRJNA918362.
